# Biological Activity of Oleanane Triterpene Derivatives Obtained by Chemical Derivatization

**DOI:** 10.3390/molecules181013003

**Published:** 2013-10-18

**Authors:** Shi-Yie Cheng, Chao-Min Wang, Hsueh-Ling Cheng, Hui-Jye Chen, Yuan-Man Hsu, Yu-Chi Lin, Chang-Hung Chou

**Affiliations:** 1 Department of Life Sciences, National University of Kaohsiung, Kaohsiung 811, Taiwan; E-Mail: shiyie@nuk.edu.tw; 2 Research Center for Biodiversity, China Medical University, Taichung 40402, Taiwan; E-Mail: wangchaomin@mail.cmu.edu.tw; 3 Department of Biological Science and Technology, National Pingtung University of Science and Technology, Pingtung 91201, Taiwan; E-Mail: hlcheng@mail.npust.edu.tw; 4 Graduate Institute of Molecular Systems Biomedicine, China Medical University, Taichung 40402, Taiwan; E-Mail: huijyechen@mail.cmu.edu.tw; 5 Department of Biological Science and Technology, China Medical University, Taichung 40402, Taiwan; E-Mail: yuanmh@mail.cmu.edu.tw; 6 Department of Life Sciences, National Cheng Kung University, Tainan 701, Taiwan; E-Mail: z10108042@email.ncku.edu.tw; 7 Graduate Institute of Ecology and Evolutionary Biology, China Medical University, Taichung 40402, Taiwan

**Keywords:** oleanane triterpenoids, * Fatsia polycarpa* Hayata, cytotoxic, anti-hepatitis B virus(HBV), antibacterial, hypoglycaemic, Wnt responsive reporter activities

## Abstract

Nine new derivatives of oleanane triterpenoids isolated from *Fatsia polycarpa* Hayata were synthesized through chemical transformations. Acetylation was effected by reaction with acetic anhydride in pyridine to afford compounds **1**–**5**, while compound **6** was obtained using 1-ethyl-3-(3-dimethylaminopropyl)carbodiimide hydrochloride (EDC·HCl) in CH_2_Cl_2_. The others derivatives **7**–**9** were obtained in reactions of the corresponding triterpenoids with EDC·HCl, 4-*N*,*N*-dimethylaminopyridine hydrochloride and 4-*N*,*N*-dimethylaminopyridine in CH_2_Cl_2_. The structures of **1**–**9** were elucidated from extensive spectroscopic and HRESIMS data, while the structure of **9** was further confirmed by X-ray diffraction analysis. The cytotoxic, anti-hepatitis B virus (HBV), antibacterial, hypoglycaemic and Wnt signaling activities of these derivatives were evaluated *in vitro*.

## 1. Introduction

Numerous oleananoids have gererated tremendous interest from the standpoint of their biological activities, such as antigiardial, anti-HIV, antihyperglycemia, anti-inflammatory, antimycobacterial, antioxidative, antitumor and cardiovascular properties [1,2,3,4,5,6,7,8,9,10]. Some of them are known to have anticarcinogenic activity in experimental animals [11]. Although the bioactivity of oleanolic acid is modest, it has been marketed in China as an oral drug for treating liver disorders in humans. It has been well recognized to possess anti-inflammatory and antihyperlipidemic activities in animals [12].

**Scheme 1:**
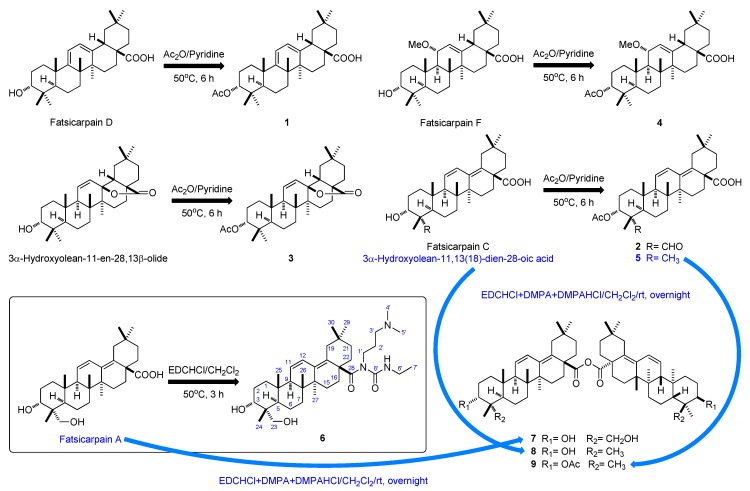
Outline of the present work (Preparation of compounds **1**–**9**).

Structure-activity relationship studies of betulinic acid and dihydrobetulinic acid derivatives have also proved that various structural modifications, for example C-3 ester substitution, may provide analogs with greatly enhanced activity [13,14]. In our previous paper we reported the isolation and characterization of seven oleananoids, named fatsicarpains A–G, with moderate cytotoxic and antibacterial activities [2]. In view of the bioactive potential of these isolated compounds, the goal of this study was to modify the two active portions of them, namely, the C-3 hydroxy group and the carboxyl group at C-28. We have thus prepared nine new derivatives **1**–**9**, as shown in Scheme 1. Compounds **1**–**5** were synthesized as acetylation products with acetic anhydride in pyridine at 50 °C for 6 h, while **6** was obtained using EDC·HCl in anhydrous CH_2_Cl_2_ at 50 °C for 3 h. Derivatives **7**–**9** were obtained in the reactions of the corresponding triterpenoids with EDC·HCl, DMAP·HCl and DMAP in CH_2_Cl_2_ at room temperature overnight. The structure elucidations of **1**–**9** were performed by NMR and HR-ESI-MS analyses. The structure of **9** was further confirmed by X-ray diffraction analyses [15].

Compounds **1**–**9** were evaluated for the *in vitro* cytotoxicity against cancer cell lines HepG2 2.2.15 (human hepatocellular carcinoma). The anti-hepatitis B virus (HBV) effects of **1**–**9**, in terms of the inhibition of hepatitis B surface antigen (HBsAg) and hepatitis B virus e antigen (HBeAg), were also measured. Moreover, bioassays were conducted for hypoglycaemic activity, and for antibacterial activity against *Helicobacter pylori*, *Bacillus cereus*, *Enterococcus faecalis*, *Escherichia coli*, *Listeria monocytogenes*, *Salmonella enterica*, *Staphylococcus aureus* and *Pseudomonas aeruginosa*. Besides, the effects of the tested compounds on the Wnt/*β*-catenin signaling, a pathway that once it is disregulated will cause many diseases including various types of cancers, were revealed by the Wnt reporter assay [16].

## 2. Results and Discussion

Compound **1** was prepared in the overnight reaction of fatsicarpain D [2] and acetic anhydride in anhydrous pyridine at 50 °C. The HRESIMS of **1** exhibited a pseudomolecular ion peak at *m/z* 519.3445 [M + Na]^+^, consistent with the molecular formula of C_32_H_48_O_4_, requiring nine degrees of unsaturation. The IR spectrum of **1** showed the diagnostic absorption band of an acetoxy functionality at 1,733 cm^−1^, which was further supported by the ^1^H-NMR signals at *δ*_H_ 2.04 (3H, s) and at *δ*_H_ 4.64 ppm for the H-3 (situated downfield from the resonance of the corresponding proton in the starting compound (*δ*_H_ 3.42 ppm) [2], and ^13^C-NMR signals at *δ*_C_ 170.8 (qC) and 21.3 (CH_3_). Additionally, acetylation induced significant downfield shifts of H-3 (Δ*δ*_H_ = 1.22 ppm) [2]. The carbonyl signal was attributed to the acetate moiety linked to C-3, as further confirmed through the crucial HMBC correlation from H-3 to the carbonyl carbon of 3-OAc. Similarly, fatsicarpain C, 3*α*-hydroxyolean-11-en-28,13*β*-olide, fatsicarpain F and 3*α*-hydroxyolean-11,13(18)-dien-28-oic acid were submitted to acetylation with Ac_2_O in pyridine at room temperature overnight to yield **2**–**5**, respectively. The NMR data revealed the presence of characteristic *O*-acetyl group signals (see Experimental). Furthermore, the structures of **2**–**5** were definitely confirmed by the crucial HMBC correlations of the acetyl carbonyl carbon signals in each case with H-3.

**Figure 1:**
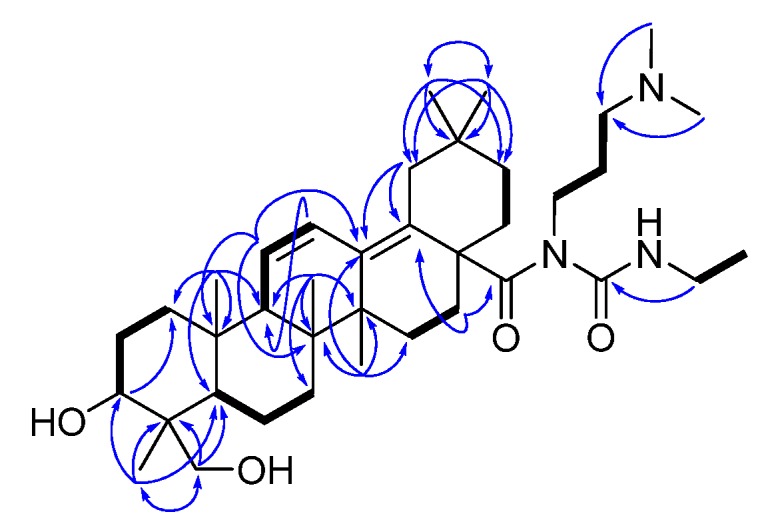
Selected ^1^H-^1^H COSY (▬) and HMBC (→) correlations of **6**.

**Figure 2:**
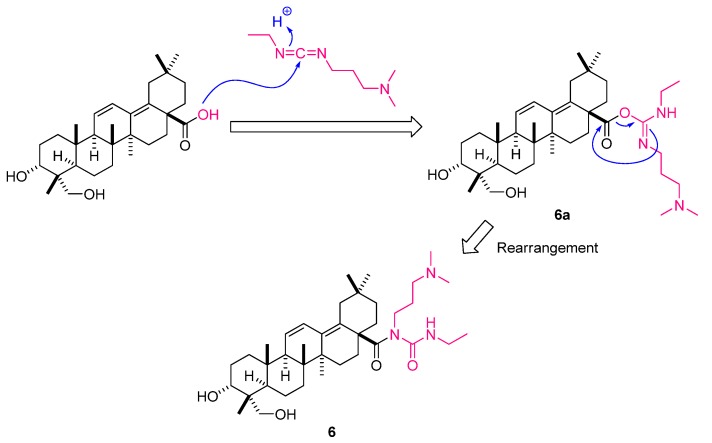
Suggested pathway for the conversion of fatsicarpain A to **6**.

*N*-(3-(Dimethylamino)propyl)-*N*-(ethylcarbamoyl)-3*α*,23-dihydroxyolean-11,13(18)-dien-28-amide (**6**), synthesized from fatsicarpain A and EDC·HCl in dry CH_2_Cl_2_ (at 50 °C for 3 h), was obtained as a white amorphous powder. Compound **6** was analyzed for the molecular formula of C_38_H_64_O_4_N_3_ by the positive HRESIMS (*m/z* 626.4901, [M+H]^+^) coupled with the ^13^C-NMR spectroscopic data (see Experimental). The NMR features of **6** were analogous to those of fatsicarpain A except that the resonances of the carboxylic acid at C-28 were replaced by those of *N*-(3-(dimethylamino)propyl)-*N*-(ethylcarbamoyl)formamide group. By interpretation of ^1^H-^1^H COSY correlations, it was possible to establish two partial structures of consecutive proton systems extending from H_2_-1' to H_2_-3' through H_2_-2', and from H_2_-6' to H_2_-7'. The crucial HMBC correlations from H_2_-16 to C-28 and from H_2_-6' to C-8' revealed the connectivity of the above partial structures (Figure 1). Consequently, the structure of **6** was unambiguously established. Moreover, the suggested pathway involves the rearrangement of the *O*-acylisourea **6a** to the stable *N*-acylurea **6**, as illustrated in Figure 2. *O*-acylisourea **6a** is unstable in anhydrous CH_2_Cl_2_ and can undergo cyclic electronic displacement (O→N acyl migration), producing the thermodynamically more stable *N*-acylurea **6** [17].

**Figure 3:**
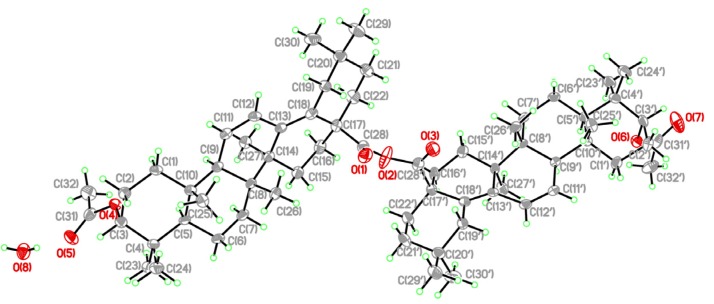
X-ray ORTEP diagram of **9**.

Compound **7**, prepared by overnight reaction of fatsicarpain A with EDC·HCl, DMAP and DMAP·HCl in CH_2_Cl_2_ at room temperature, was obtained as a white amorphous powder. It was analyzed for the molecular formula of C_60_H_90_O_7_ by the positive HRESIMS (*m/z* 945.6595, [M+Na]^+^) coupled with its ^13^C-NMR spectroscopic data (see Experimental). The characteristic pattern for a noncyclic and saturated anhydride in the IR spectrum of **7** is the appearance of two strong bands at 1,793 and 1,772 cm^−1^, and this was further supported by the ^13^C-NMR signals at *δ*_C_ 172.7 (qC). Anhydride coupling induced significant upfield shifts of C-28 (Δ*δ*_C_ = 7.3 ppm) [2]. The position of the anhydride group was confirmed by the HMBC correlations from H_2_-16 to C-28. In the same manner, compounds **8** and **9** were prepared by anhydride coupling of 3*α*-hydroxyolean-11,13(18)-dien-28-oic acid and **5**, respectively. The IR spectra and ^13^C-NMR data revealed the presence of characteristic anhydride group signals (see Experimental). In addition, compound **9** was fully characterized by the X-ray analysis (Figure 3) which confirmed the structure and presence of anhydride group.

Of four million patients worldwide infected with hepatitis B virus (HBV), about 20% are expected to develop chronic hepatitis, liver cirrhosis, or hepatocarcinoma [18]. Anti-HBV effects of compounds **1**–**9** were screened *in vitro* using the human hepatocellular carcinoma (HepG2 2.2.15) cell model system with fluorouracil as a positive control (Table 1). Preliminary cytotoxicity screening revealed that **1**–**6** exhibited inhibition effects on HBV replicated DNA level in the IC_50_ values of 6.5, 17.9, 38.5, 24.1, 9.3 and 5.3 *μ*M, respectively. The esterification of the hydroxy group at C-3 increased the resultant cytotoxicity against HepG2 2.2.15, as shown for the acetylated derivatives **1**–**5**. By far, compound **6** exhibited the highest potency against HepG2 2.2.15. Particularly, compounds **7**–**9**, possessing an anhydride group, showed no significant activity against HepG2 2.2.15 (IC_50_ > 50 *μ*M), which confirmed that the carboxylic acid at C-28 is essential to their cytotoxicity. After varying its structure at C-3 and C-28 positions, we found **1**–**6** to be more potent than the parent compounds (Table 1), and these findings are in accordance with the available literature data concering structure−activity relationship [13,14]. However, hepatitis B surface antigen (HBsAg) and hepatitis B virus e antigen (HBeAg) in HepG2 2.2.15 cells were not significantly inhibited by the tested compounds.

**Table 1:** Cytotoxicity data of compounds **1**–**9**, fatsicarpains A, C, D, F, 3*α*-hydroxyolean-11-en-28,13*β*-olide and 3*α*-hydroxyolean-11,13(18)-dien-28-oic acid.

	IC_50_ (μg/mL)		
Compounds	HepG2 2.2.15	HBsAg	HBeAg
**1**	6.5	>50	>50
**2**	17.9	>50	>50
**3**	38.5	>50	>50
**4**	24.1	>50	>50
**5**	9.3	>50	>50
**6**	5.3	>50	>50
**7**	>50	>50	>50
**8**	>50	>50	>50
**9**	>50	>50	>50
Fatsicarpain A	18.9	>50	>50
Fatsicarpain C	16.7	>50	>50
Fatsicarpain D	28.8	>50	>50
Fatsicarpain F	23.9	>50	>50
3 *á*-Hydroxyolean-11-en-28,13*â*-olide	>50	>50	>50
3 *á*-Hydroxyolean-11,13(18)-dien-28-oic acid	>50	>50	>50

*Helicobacter* *pylori* infection is associated with an increased risk for development of duodenal ulcers, gastric ulcers, gastric adenocarcinomas and gastric lymphomas. However, as other bacterial pathogens, antibiotic resistance to *H.* *pylori* is an increasing problem for eradicating infection [19]. Therefore, finding a safe and efficient treatment to decrease the need or even replace antibiotics for eradicating *H.* *pylori* infection in human becomes necessary and an important task. Preliminary anti-*H.* *pylori* activity revealed that compound **2** exhibited moderate antibacterial activity with a minimum bactericidal concentrations (MBC) of 128 *μ*g/mL. With the exception of the above observations, the obtained negative results showed that compounds **1**, **3**–**5** and **7**–**9** exhibited no discernible activity (MBC > 128 *μ*g/mL) (Table 2).

In the past decades, bacterial resistance to the antibiotics has emerged a serious global problem in human and veterinary medicine. The abuse of antibiotics for non-perscription application has accelerated the generation of superbacteria which makes a critical issue. According to a previous report from 1991 to 2000, the *Bacillus cereus* played the leading role of outbreak case of food-borne pathogens (41.2%, 113 of 171 outbreaks), followed by *Staphylococcus aureus* (17.9%) and *Vibrio parahaemolyticus* (15.7%) in central Taiwan [20]. Also, the modifiction at C-3 position of betulinic acid, oleanolic acid and ursolic acid increased antimycobacterial activity aganist *Mycobacterium tubereulosis* [21]. For that reason the antibacterial activities of new modified oleanane-type derivatives **1**–**9** were evalutaed against seven bacteria: *B. cereus*, *E. faecalis*, *E. coli*, *L. monocytogenes*, *S. enterica*, *S. aureus* and *P. aeruginosa* and compared with the activity of parent compounds (Table 2) [2]. As expected, the C-3 acetylated derivatives **2**, **5** and **6** exhibited more potent than the parent compounds. Additionally, compounds **2**, **5** and **6** revealed greater antibacterial potential than the positive control (ampicillin) against *B. cereus*. Particularly, compounds **5** and **6** showed significant antibacterial activity against *B. cereus* with MIC values at 2 and 8 *µ*g/mL, respectively. Only **6** showed specific antibacterial activity against *S. aureus* and *L*. *monocytogenes* with MIC values at 16 and 32 *µ*g/mL, respectively. It was noteworthy to mention that modification of the functional group at C-28 from carboxylic acid to *N*-(3-(dimethylamino)propyl)-*N*-(ethylcarbamoyl)formamide moiety increased the antibacterial activity against *L*. *monocytogenes* significantly. Moreover, it is thought that coupling of two active compounds would generate more activity, but anhydride derivatives **7**–**9** did not exhibit significant antibacterial activity against all tested pathogens. The present result suggested that the presence of the C-28 carboxylic acid moiety is important for significant activity against *B. cereus*. However, none of the tested compounds had significant activity against Gram-negative pathogens, *E. coli*, *S. enterica* and *P. aeruginosa*.

**Table 2:** Antibacterial activity of **1**–**9**, fatsicarpains A, C, D, F, 3*α*-hydroxyolean-11-en-28,13*β*-olide (X) and 3*α*-hydroxyolean-11,13(18)-dien-28-oic acid (Y).

	Minimum Bactericidal Concentrations (MBC) (μg/mL)														
Pathogens	A	C	D	F	X	Y	1	2	3	4	5	6	7	8	9
*H. pylori*	64	64	64	64	>128	>128	>128	128	>128	>128	>128		>128	>128	>128
	Minimum Inhibitory Concentrations (MIC) (μg/mL)														
*S* *.* * aureus*	>128	64	>128	>128	>128	>128	>128	128	>128	>128	>128	16	>128	>128	>128
*E* *.* * faecalis*	>128	>128	>128	>128	>128	>128	>128	128	>128	>128	>128	32	128	128	128
*L* *.* * mo* *nocytogenes*	>128	>128	>128	>128	>128	>128	>128	>128	>128	>128	>128	32	>128	>128	>128
*B* *.* * cereus*	32	32	8	2	>128	>128	>128	16	>128	>128	2	8	>128	>128	>128
*E* *.* * coli*	>128	>128	>128	>128	>128	>128	>128	>128	>128	>128	>128	>128	>128	>128	>128
*S* *.* * enterica*	>128	>128	>128	>128	>128	>128	>128	>128	>128	>128	>128	>128	>128	>128	>128
*P* *.* * aeruginosa*	>128	>128	>128	>128	>128	>128	>128	>128	>128	>128	>128	>128	>128	>128	>128

Ampicillin was used as a positive control, and it showed antibacterial activity against *S. Aureus*, *E. Faecalis*, *L. monocytogenes*, *B. cereus*, *E. coli*, *S. enterica*, and *P. aeruginosa* with MIC values of 8, 2, 1, 128, 4, 1 and 512 µg/mL, respectively.

**Figure 4:**
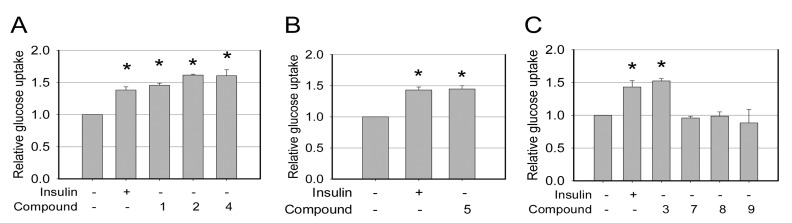
Glucose uptake assays for cells treated with compounds (20 μM). A, assays for **1**, **2** and **4**; B, assay for **5**; C, assays for **3**, **7**, **8** and **9**. The total amount of medium glucose consumed by the cells between 0 to 5 h of treatment was calculated, and data expressed as relative glucose uptake *versus* control (cells with no treatment). Data represent the mean ± standard deviation of triplicate. * *p* < 0.05 *versus* control by two-way ANOVA.

The hypoglycaemic activities were determined only for the modified compounds **1**–**5** and **7**–**9**, the exception being **6** due to insufficient quantities for testing. The hypoglycaemic activities were tested using cell-based screening method, in which glucose uptake of cells treated with the new compound is quantified and compared with that of untreated cells and cells stimulated with insulin [22,23]. It was found that compounds **1**–**5** but not **7**–**9** enhanced glucose uptake of treated cells compared with untreated, and that their effects were similar to that of insulin (Figure 4), indicating that compounds **1**–**5** possess insulin-like hypoglycaemic activities that can promote the glucose uptake of cells. However, the underlying mechanism is not clear. Whether it involves the activation of the insulin signaling pathway requires further investigation.

**Figure 5:**
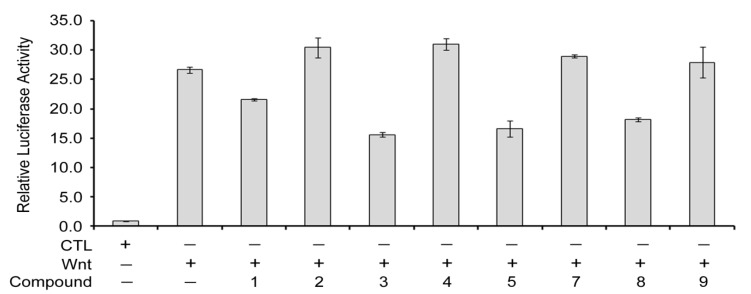
The effect of oleanane triterpene derivatives on β-catenin/TCF-mediated luciferase activities in P19 cells. Cells were transfected with Wnt reporter pGL3-OT and normalization vector pTK-Renilla, and treated with control-conditioned medium (CTL), Wnt3a-conditioned medium (Wnt), or different compounds of oleanane triterpene derivatives in Wnt3a-conditioned medium (compounds **1**–**5** and **7**–**9**) for 20 h, then cell lysates were harvested for dual luciferase activity assays. Each bar is the mean ±S.D. Each experiment was performed in triplicate.

Wnts proteins are secreted lipoglycoproteins that function as signaling molecules to regulate embryonic development and tissue homeostatis [24]. Aberrant Wnt signaling can cause an array of human diseases, including schizophrenia, pulmonary fibrosis, rheumatoid arthritis [25,26], osteoporosis, tetraamelia syndrome, neurodegenerative diseases and various cancers [27,28,29,30,31]. To know more about the biological effects of **1**–**5** and **7**–**9**, these compounds were tested on the inhibition of canonical Wnt signaling using the TCF/*β*-catenin-mediated luciferase activity assay [16]. Compound **6** again was not tested due to paucity of the sample. As compared to the assay performed in Wnt-3a conditioned medium without any drug treatment (set as 100%), compounds **1**, **3**, **5** and **8** at 1 *μ*M of concentration specifically inhibited the Wnt signaling by 20%, 40%, 38% and 32%, respectively, while **2**, **4**, **7** and **9** were not effective (Figure 5). These data showed that Wnt signaling is sensitive to **1**, **3**, **5** and **8** and also suggested the potential use of these compounds for the therapy of Wnt-related diseases in the future.

## 3. Experimental

### 3.1. General

Optical rotations were determined with a JASCO P2000 digital polarimeter. Ultraviolet (UV) and infrared (IR) spectra were obtained on JASCO V-650 and JASCO FT/IR-4100 spectrophotometers, respectively. The NMR spectra were recorded on a Varian Unity INOVA 600 FT-NMR spectrometer (600 MHz for ^1^H; 150 MHz for ^13^C, respectively). Chemical shifts were reported using residual CDCl_3_ (*δ*_H_ 7.26 and *δ*_C_ 77.0 ppm) as internal standard. High-resolution ESIMS spectra were obtained on a LTQ Orbitrap XL (Thermo Fisher Scientific) spectrometer. The crystallographic data were collected on a Bruker D8 Discover SSS X-ray diffractometer equipped with a closed molybdenum tube generator and parabolic Göbel mirror. Silica gel 60 (Merck, 230–400 mesh), LiChroprep RP-18 (Merck, 40–63 *μ*m) and Sephadex LH-20 (Amersham Pharmacia Biotech.) were used for column chromatography (CC). Pre-coated silica gel plates (Merck, Kieselgel 60 F_254_, 0.25 mm) and pre-coated RP-18 F_254s_ plates (Merck) were used for analytical thin-layer chromatography (TLC) analyses. High-performance liquid chromatography (HPLC) was carried out using a Hitachi L-2130 pump equipped with a Hitachi L-2420 UV-vis detector at 220 nm and a semi-preparative reversed-phase column (Merck, Hibar Purospher RP-18e, 5 *μ*m, 250 × 10 mm).

### 3.2. Plant Material

Leaves and twigs of *Fatsia polycarpa* (7.1 kg) were collected at Hohuan Mountain (2,105 m elevation), Taiwan, in November 2009, and identified by one of the authors (C.-H. C.). A voucher specimen (FPL) was deposited in the Research Center for Biodiversity, China Medical University, Taiwan.

### 3.3. Acetylation

Fatsicarpain D (2.0 mg) was dissolved in pyridine (0.5 mL) and allowed to react overnight at room temperature with acetic anhydride (one drop). Then, the reaction was quenched by the addition of 1.0 mL of H_2_O, followed by extraction with EtOAc (3 × 1.0 mL). The EtOAc extracts were combined and evaporated. The resulting residue was subjected to a short silica gel column using *n*-hexane–EtOAc (3:1) to yield an acetylated product **1** (1.9 mg). Additionally, fatsicarpain C (3.0 mg), 3*α*-hydroxyolean-11-en-28,13*β*-olide (3.0 mg), fatsicarpain F (3.0 mg) and 3*α*-hydroxyolean-11,13(18)-dien-28-oic acid (3.0 mg) were subjected to acetylation, and chromatographic purification of the product according to the same procedure mentioned under acetylation of fatsicarpain D, to give **2** (2.9 mg), **3** (2.8 mg), **4** (2.7 mg) and **5** (2.9 mg), respectively.

*3α-Acetoxyolean-9,12-dien-28-oic acid* (**1**). Yield 91%. white amorphous powder; [*α*]^25^_D_ +75 (*c* = 0.1, CHCl_3_); UV (MeOH) *λ*_max_ (log *ε*) 212 (4.40), 274 (3.85) nm; IR (KBr) ν_max_ 3,458, 2,941, 1,733, 1,700, 1,455, 1,374, 1,245, 758 cm**^−^**^1^; ^1^H-NMR (CDCl_3_, 600 MHz) *δ*_H_ 1.59 (1H, m, H-1*α*), 1.76 (1H, m, H-1*β*), 1.73 (1H, m, H-2*α*), 1.94 (1H, m, H-2*β*), 4.64 (1H, br t, *J* = 3.0 Hz, H-3), 1.31 (1H, m, H-5), 1.50 (2H, m, H-6), 1.72 (1H, m, H-7*α*), 1.32 (1H, m, H-7*β*), 5.60 (1H, d, *J* = 6.0 Hz, H-11), 5.58 (1H, d, *J* = 6.0 Hz, H-12), 1.16 (1H, m, H-15*α*), 1.83 (1H, m, H-15*β*), 1.99 (1H, m, H-16*α*), 1.68 (1H, m, H-16*β*), 2.98 (1H, dd, *J* = 13.8, 4.2 Hz, H-18), 1.62 (1H, m, H-19*α*), 1.20 (1H, m, H-19*β*), 1.23 (1H, m, H-21*α*), 1.36 (1H, m, H-21*β*), 1.79 (1H, m, H-22*α*), 1.66 (1H, m, H-22*β*), 0.89 (3H, s, H-23), 0.91 (1H, s, H-24), 1.19 (3H, s, H-25), 0.99 (3H, s, H-26), 1.08 (3H, s, H-27), 0.95 (3H, s, H-29), 0.90 (3H, s, H-30), 2.04 (3H, s, 3-OAc); ^13^C-NMR (CDCl_3_, 150 MHz) *δ*_C_ 32.2 (t, C-1), 23.3 (t, C-2), 77.8 (d, C-3), 36.8 (s, C-4), 46.0 (d, C-5), 18.0 (t, C-6), 31.8 (t, C-7), 42.4 (s, C-8), 154.8 (s, C-9), 38.7 (s, C-10), 115.2 (d, C-11), 120.7 (d, C-12), 144.7 (s, C-13), 40.8 (s, C-14), 26.9 (t, C-15), 23.6 (t, C-16), 45.9 (s, C-17), 39.4 (d, C-18), 45.9 (t, C-19), 30.6 (s, C-20), 33.7 (t, C-21), 32.1 (t, C-22), 27.8 (q, C-23), 21.9 (q, C-24), 24.8 (q, C-25), 20.4 (q, C-26), 20.1 (q, C-27), 182.8 (s, C-28), 23.5 (q, C-29), 32.9 (q, C-30), 170.8 (s, 3-OAc), 21.3 (q, 3-OAc); ESIMS *m/z* 519 [M+Na]^+^; HRESIMS *m/z* 519.3445 [M+Na]^+^ (Calcd for C_32_H_48_O_4_Na, 519.3445).

*3α-Acetoxy-24-formylolean-11,13(18)-dien-28-oic acid* (**2**). Yield 85%. white amorphous powder; [*α*]^25^_D_ −64 (*c* = 0.1, CHCl_3_); UV (MeOH) *λ*_max_ (log *ε*) 235 (4.08), 247 (3.78), 263 (3.55) nm; IR (KBr) ν_max_ 3,454, 2,941, 2,861, 1,734, 1,698, 1,450, 1,373, 1,242, 759 cm**^−^**^1^; ^1^H-NMR (CDCl_3_, 600 MHz) *δ*_H_ 1.33 (1H, m, H-1*α*), 1.51 (1H, m, H-1*β*), 1.78 (1H, m, H-2*α*), 1.93 (1H, m, H-2*β*), 4.87 (1H, br s, H-3), 2.01 (1H, m, H-5), 1.50 (2H, m, H-6), 1.33 (1H, m, H-7*α*), 1.25 (1H, m, H-7*β*), 2.20 (1H, br s, H-9), 5.64 (1H, d, *J* = 10.2 Hz, H-11), 6.47 (1H, dd, *J* = 10.2, 3.0 Hz, H-12), 1.10 (1H, m, H-15*α*), 1.70 (1H, m, H-15*β*), 2.01 (1H, m, H-16*α*), 1.71 (1H, m, H-16*β*), 2.54 (1H, d, *J* = 14.4 Hz, H-19*α*), 1.69 (1H, d, *J* = 14.4 Hz, H-19*β*), 1.32 (1H, m, H-21*α*), 1.39 (1H, m, H-21*β*), 2.28 (1H, m, H-22*α*), 1.40 (1H, m, H-22*β*), 1.08 (3H, s, H-23), 9.41 (1H, s, H-24), 0.97 (3H, s, H-25), 0.82 (3H, s, H-26), 1.06 (3H, s, H-27), 0.81 (3H, s, H-29), 0.96 (3H, s, H-30), 2.06 (3H, s, 3-OAc); ^13^C-NMR (CDCl_3_, 150 MHz) *δ*_C_ 31.8 (t, C-1), 22.2 (t, C-2), 74.6 (d, C-3), 51.1 (s, C-4), 43.9 (d, C-5), 20.3 (t, C-6), 31.9 (t, C-7), 41.2 (s, C-8), 53.9 (d, C-9), 36.1 (s, C-10), 126.2 (d, C-11), 125.5 (d, C-12), 136.8 (s, C-13), 42.2 (s, C-14), 24.8 (t, C-15), 32.6 (t, C-16), 48.0 (s, C-17), 131.5 (s, C-18), 40.5 (t, C-19), 32.4 (s, C-20), 36.8 (t, C-21), 35.4 (t, C-22), 14.0 (q, C-23), 205.9 (d, C-24), 17.8 (q, C-25), 16.6 (q, C-26), 19.9 (q, C-27), 180.9 (s, C-28), 24.0 (q, C-29), 32.2 (q, C-30), 170.0 (s, 3-OAc), 21.2 (q, 3-OAc); ESIMS *m/z* 533 [M + Na]^+^; HRESIMS *m/z* 533.3207 [M + Na]^+^ (Calcd for C_32_H_46_O_5_Na, 533.3237).

*3α-Acetoxyolean-11-en-28,13β-olide* (**3**). Yield 82%. white amorphous powder; [*α*]^25^_D_ +52 (*c* = 0.1, CHCl_3_); IR (KBr) *v*_max_ 2,941, 1,750, 1,736, 1,456, 1,376, 1,247, 1,158, 756 cm**^−^**^1^; ^1^H-NMR (CDCl_3_, 600 MHz) *δ*_H_ 1.17 (1H, m, H-1*α*), 1.62 (1H, m, H-1*β*), 1.63 (1H, m, H-2*α*), 1.95 (1H, m, H-2*β*), 4.65 (1H, t, *J* = 2.4 Hz, H-3), 1.24 (1H, m, H-5), 1.50 (2H, m, H-6), 1.45 (1H, m, H-7*α*), 1.26 (1H, m, H-7*β*), 2.01 (1H, br s, H-9), 6.05 (1H, d, *J* = 10.2 Hz, H-11), 5.42 (1H, dd, *J* = 10.2, 3.0 Hz, H-12), 1.21 (1H, m, H-15*α*), 1.73 (1H, m, H-15*β*), 2.13 (1H, td, *J* = 13.2, 6.6 Hz, H-16*α*), 1.36 (1H, m, H-16*β*), 2.08 (1H, m, H-18), 1.82 (1H, t, *J* = 13.2 Hz, H-19*α*), 1.36 (1H, m, H-19*β*), 1.32 (1H, m, H-21*α*), 1.36 (1H, m, H-21*β*), 1.65 (1H, m, H-22*α*), 1.69 (1H, m, H-22*β*), 0.85 (3H, s, H-23), 0.90 (3H, s, H-24), 0.93 (3H, s, H-25), 1.07 (3H, s, H-26), 1.12 (3H, s, H-27), 0.88 (3H, s, H-29), 0.97 (3H, s, H-30), 2.08 (3H, s, 3-OAc); ^13^C-NMR (CDCl_3_, 150 MHz) *δ*_C_ 33.5 (t, C-1), 22.6 (t, C-2), 78.1 (d, C-3), 36.7 (s, C-4), 49.6 (d, C-5), 17.4 (t, C-6), 30.9 (t, C-7), 41.4 (s, C-8), 52.9 (d, C-9), 36.4 (s, C-10), 135.8 (d, C-11), 126.8 (d, C-12), 89.8 (s, C-13), 41.7 (s, C-14), 25.3 (t, C-15), 21.3 (t, C-16), 44.0 (s, C-17), 50.5 (d, C-18), 37.3 (t, C-19), 31.4 (s, C-20), 34.3 (t, C-21), 27.1 (t, C-22), 27.6 (q, C-23), 21.2 (q, C-24), 17.7 (q, C-25), 19.0 (q, C-26), 18.5 (q, C-27), 180.1 (s, C-28), 23.5 (q, C-29), 33.2 (q, C-30), 170.7 (s, 3-OAc), 21.4 (q, 3-OAc); ESIMS *m/z* 519 [M + Na]^+^; HRESIMS *m/z* 519.3422 [M + Na]^+^ (Calcd for C_32_H_48_O_4_Na, 519.3445).

*3α-Acetoxy-11α-methoxyolean-12-en-28-oic acid* (**4**). Yield 83%. white amorphous powder; [*α*]^25^_D_ −76 (*c* = 0.1, CHCl_3_); IR (KBr) *v*_max_ 2,941, 1,737, 1,697, 1,450, 1,373, 1,243, 1,110, 756 cm**^−^**^1^; ^1^H-NMR (CDCl_3_, 600 MHz) *δ*_H_ 1.49 (1H, m, H-1*α*), 1.60 (1H, m, H-1*β*), 1.61 (1H, m, H-2*α*), 2.06 (1H, m, H-2*β*), 4.62 (1H, br s, H-3), 1.24 (1H, m, H-5), 1.46 (2H, m, H-6), 1.50 (1H, m, H-7*α*), 1.22 (1H, m, H-7*β*), 1.79 (1H, d, *J* = 8.4 Hz, H-9), 3.74 (1H, dd, *J* = 8.4, 3.6 Hz, H-11), 5.53 (1H, d, *J* = 3.6 Hz, H-12), 1.18 (1H, m, H-15*α*), 2.01 (1H, m, H-15*β*), 2.03 (1H, m, H-16*α*), 1.63 (1H, m, H-16*β*), 2.87 (1H, dd, *J* = 13.2, 3.6 Hz, H-18), 1.61 (1H, m, H-19*α*), 1.19 (1H, m, H-19*β*), 1.24 (1H, m, H-21*α*), 1.37 (1H, m, H-21*β*), 1.80 (1H, m, H-22*α*), 1.62 (1H, m, H-22*β*), 0.85 (3H, s, H-23), 0.89 (3H, s, H-24), 1.03 (3H, s, H-25), 0.78 (3H, s, H-26), 1.26 (3H, s, H-27), 0.96 (3H, s, H-29), 0.93 (3H, s, H-30), 2.07 (3H, s, 3-OAc); ^13^C-NMR (CDCl_3_, 150 MHz) *δ*_C_ 34.3 (t, C-1), 29.4 (t, C-2), 78.4 (d, C-3), 36.5 (s, C-4), 49.9 (d, C-5), 18.1 (t, C-6), 32.8 (t, C-7), 42.8 (s, C-8), 53.0 (d, C-9), 38.2 (s, C-10), 76.0 (d, C-11), 121.6 (d, C-12), 148.3 (s, C-13), 41.9 (s, C-14), 27.8 (t, C-15), 22.8 (t, C-16), 46.0 (s, C-17), 40.7 (d, C-18), 45.6 (t, C-19), 30.7 (s, C-20), 33.7 (t, C-21), 32.2 (t, C-22), 28.0 (q, C-23), 21.9 (q, C-24), 16.8 (q, C-25), 18.7 (q, C-26), 25.5 (q, C-27), 180.4 (s, C-28), 23.5 (q, C-29), 33.0 (q, C-30), 170.9 (s, 3-OAc), 21.4 (q, 3-OAc); ESIMS *m/z* 551 [M + Na]^+^; HRESIMS *m/z* 551.3686 [M + Na]^+^ (Calcd for C_33_H_52_O_5_Na, 551.3707).

*3α-Acetoxy olean-11,13(18)-dien-28-oic acid* (**5**). Yield 85%. white amorphous powder; [*α*]^25^_D_ −51 (*c* = 0.1, CHCl_3_); UV (MeOH) *λ*_max_ (log *ε*) 232 (4.10), 245 (3.68), 264 (3.65) nm; IR (KBr) *v*_max_ 3,464, 2,941, 1,731, 1,696, 1,456, 1,376, 1,247, 756 cm**^−^**^1^; ^1^H-NMR (CDCl_3_, 600 MHz) *δ*_H_ 1.25 (1H, m, H-1*α*), 1.64 (1H, m, H-1*β*), 1.64 (1H, m, H-2*α*), 1.96 (1H, m, H-2*β*), 4.66 (1H, br s, H-3), 1.30 (1H, m, H-5), 1.50 (2H, m, H-6), 1.38 (1H, m, H-7*α*), 1.34 (1H, m, H-7*β*), 2.05 (1H, br s, H-9), 5.65 (1H, d, *J* = 10.2 Hz, H-11), 6.43 (1H, dd, *J* = 10.2, 2.4 Hz, H-12), 1.09 (1H, m, H-15*α*), 1.72 (1H, m, H-15*β*), 2.00 (1H, m, H-16*α*), 1.72 (1H, m, H-16*β*), 2.54 (1H, d, *J* = 14.4 Hz, H-19*α*), 1.67 (1H, d, *J* = 14.4 Hz, H-19*β*), 1.29 (1H, m, H-21*α*), 1.40 (1H, m, H-21*β*), 2.27 (1H, m, H-22*α*), 1.40 (1H, m, H-22*β*), 0.89 (3H, s, H-23), 0.85 (3H, s, H-24), 0.93 (3H, s, H-25), 0.80 (3H, s, H-26), 1.02 (3H, s, H-27), 0.81 (3H, s, H-29), 0.95 (3H, s, H-30), 2.09 (3H, s, 3-OAc); ^13^C-NMR (CDCl_3_, 150 MHz) *δ*_C_ 33.3 (t, C-1), 22.6 (t, C-2), 78.3 (d, C-3), 36.7 (s, C-4), 49.7 (d, C-5), 18.0 (t, C-6), 32.2 (t, C-7), 40.8 (s, C-8), 54.1 (d, C-9), 36.7 (s, C-10), 127.0 (d, C-11), 125.2 (d, C-12), 137.0 (s, C-13), 42.0 (s, C-14), 24.8 (t, C-15), 32.6 (t, C-16), 48.0 (s, C-17), 130.9 (s, C-18), 40.4 (t, C-19), 32.5 (s, C-20), 36.8 (t, C-21), 35.5 (t, C-22), 27.7 (q, C-23), 21.3 (q, C-24), 17.8 (q, C-25), 16.4 (q, C-26), 19.9 (q, C-27), 181.9 (s, C-28), 24.0 (q, C-29), 32.2 (q, C-30), 170.9 (s, 3-OAc), 21.4 (q, 3-OAc); ESIMS *m/z* 519 [M + Na]^+^; HRESIMS *m/z* 519.3422 [M+Na]^+^ (Calcd for C_32_H_48_O_4_Na, 519.3445).

### 3.4. Acylation

To a solution of fatsicarpain A (5.0 mg) in CH_2_Cl_2_ (1.0 mL) EDC·HCl (1.0 mg) was added. The solution was stirred for 3 h at 50 °C. After the completion of the reaction the solvent was evaporated under reduced pressure to give a crude product which was subjected to a short silica gel column eluting with *n*-hexane–EtOAc (1:1) to yield **6** (3.2 mg).

*N-(3-(Dimethylamino)propyl)-N-(ethylcarbamoyl)-3α,23-dihydroxyolean-11,13(18)-dien-28-amide* (**6**). Yield 48%. white amorphous powder; [*α*]^25^_D_ –13 (*c* = 0.1, CHCl_3_); UV (MeOH) *λ*_max_ (log *ε*) 236 (4.11), 247 (3.88), 262 (3.74) nm; IR (KBr) *v*_max_ 3,414, 2,933, 1,696, 1,686, 1,651, 1,463, 1,363, 1,256, 1,225, 1,051, 756 cm**^−^**^1^; ^1^H-NMR (CDCl_3_, 600 MHz) *δ*_H_ 1.39 (1H, m, H-1*α*), 1.64 (1H, m, H-1*β*), 1.53 (1H, m, H-2*α*), 2.02 (1H, m, H-2*β*), 3.72 (1H, br s, H-3), 1.74 (1H, m, H-5), 1.45 (2H, m, H-6), 1.42 (2H, m, H-7), 2.14 (1H, br s, H-9), 5.65 (1H, d, *J* = 10.2 Hz, H-11), 6.43 (1H, d, *J* = 10.2, 3.0 Hz, H-12), 1.07 (1H, m, H-15*α*), 1.65 (1H, m, H-15*β*), 2.63 (1H, m, H-16*α*), 1.64 (1H, m, H-16*β*), 2.53 (1H, d, *J* = 14.4 Hz, H-19*α*), 1.47 (1H, d, *J* = 14.4 Hz, H-19*β*), 1.28 (1H, m, H-21*α*), 1.42 (1H, m, H-21*β*), 2.41 (1H, m, H-22*α*), 1.26 (1H, m, H-22*β*), 3.55 (1H, d, *J* = 11.4 Hz, H-23a), 3.42 (1H, d, *J* = 11.4 Hz, H-23b), 0.70 (3H, s, H-24), 0.95 (3H, s, H-25), 0.89 (3H, s, H-26), 1.00 (3H, s, H-27), 0.83 (3H, s, H-29), 0.92 (3H, s, H-30), 4.08 (1H, m, H-1'a), 3.38 (1H, m, H-1'b), 2.02 (1H, m, H-2'a), 1.75 (1H, m, H-2'b), 2.64 (1H, m, H-3'a), 2.33 (1H, m, H-3'b), 2.30 (3H, s, H-4'), 2.30 (3H, s, H-5'), 3.28 (1H, m, H-6'a), 3.22 (1H, m, H-6'b), 1.16 (3H, t, J = 6.6 Hz, H-7'); ^13^C-NMR (CDCl_3_, 150 MHz) *δ*_C_ 32.4 (t, C-1), 26.2 (t, C-2), 76.7 (d, C-3), 40.4 (s, C-4), 42.6 (d, C-5), 18.0 (t, C-6), 32.0 (t, C-7), 40.8 (s, C-8), 54.1 (d, C-9), 36.7 (s, C-10), 126.8 (d, C-11), 125.5 (d, C-12), 135.9 (s, C-13), 41.5 (s, C-14), 24.2 (t, C-15), 30.4 (t, C-16), 52.0 (s, C-17), 134.1 (s, C-18), 40.7 (t, C-19), 33.0 (s, C-20), 37.7 (t, C-21), 29.7 (t, C-22), 71.3 (t, C-23), 17.4 (q, C-24), 18.0 (q, C-25), 16.7 (q, C-26), 19.5 (q, C-27), 178.2 (s, C-28), 24.7 (q, C-29), 31.9 (q, C-30), 45.5 (t, C-1'), 24.7 (t, C-2'), 55.2 (t, C-3'), 44.0 (q, C-4'), 44.0 (q, C-5'), 35.6 (t, C-6'), 14.6 (q, C-7'); ESIMS *m/z* 626 [M + H]^+^; HRESIMS *m/z* 626.4901 [M + H]^+^ (Calcd for C_38_H_64_O_4_N_3_, 626.4891).

### 3.5. Anhydride Coupling Esterification

To fatsicarpain A (2.0 mg) in CH_2_Cl_2_ (1.0 mL) at room temperature were successively added DMAP (1.0 mg), DMAP·HCl (0.1 mg) and EDC·HCl (1.0 mg) and the mixture was allowed to react overnight. The reaction was quenched by water, followed by extraction with EtOAc (3 × 1.5 mL). The EtOAc extract was successively washed with 5% aqueous HCl, saturated aqueous NaHCO_3_, and brine. The organic layer was dried over anhydrous MgSO_4_ and evaporated to give a crude product, which was subjected to a short silica gel column eluting with *n*-hexane–EtOAc (3:1) to yield **7** (1.7 mg). Similarly, 3*α*-hydroxyolean-11,13(18)-dien-28-oic acid (2.0 mg) and 3*α*-acetoxyolean-11,13(18)-dien-28-oic acid (**5**) (2.0 mg) in CH_2_Cl_2_ (1.0 mL) were submitted to anhydride coupling esterification with DMAP, DMAP·HCl and EDC·HCl at room temperature overnight to yield **8** (1.8 mg) and **9** (1.8 mg), respectively.

*3α,23-Dihydroxyolean-11,13(18)-dien-28-oic anhydride* (**7**). Yield 42%. white amorphous powder; [*α*]^25^_D_ –24 (*c* 0.2, CHCl_3_); UV (MeOH) *λ*_max_ (log *ε*) 237 (4.16), 248 (3.90), 264 (3.73) nm; IR (KBr) *v*_max_ 3,446, 2,937, 1,793, 1,772, 1,734, 1,457, 1,374, 1,243, 997 cm^−1^; ^1^H-NMR (CDCl_3_, 600 MHz) *δ*_H_ 1.41 (2H, m, H-1*α* and H-1'*α*), 1.69 (2H, m, H-1*β* and H-1'*β*), 1.53 (2H, m, H-2*α* and H-2'*α*), 2.01 (2H, m, H-2*β* and H-2'*β*), 3.73 (2H, br s, H-3 and H-3'), 1.76 (2H, br d, *J* = 10.8 Hz, H-5 and H-5'), 1.47 (4H, m, H-6 and H-6'), 1.43 (4H, m, H-7 and H-7'), 2.13 (2H, br s, H-9 and H-9'), 5.69 (2H, d, *J* = 10.2 Hz, H-11 and H-11'), 6.44 (2H, dd, *J* = 10.2, 2.4 Hz, H-12 and H-12'), 1.09 (2H, m, H-15*α* and H-15'*α*), 1.64 (2H, m, H-15*β* and H-15'*β*), 2.05 (2H, m, H-16*α* and H-16'*α*), 1.71 (2H, m, H-16*β* and H-16'*β*), 2.54 (2H, d, *J* = 14.4 Hz, H-19*α* and H-19'*α*), 1.80 (2H, dd, *J* = 14.4, 1.8 Hz, H-19*β* and H-19'*β*), 1.39 (2H, m, H-21*α* and H-21'*α*), 1.33 (2H, m, H-21*β* and H-21'*β*), 2.21 (2H, m, H-22*α* and H-22'*α*), 1.47 (2H, m, H-22*β* and H-22'*β*), 3.55 (2H, d, *J* = 11.4 Hz, H-23a and H-23'a), 3.43 (2H, d, *J* = 11.4 Hz, H-23b and H-23'b), 0.70 (6H, s, H-24 and H-24'), 0.94 (6H, s, H-25 and H-25'), 0.79 (6H, s, H-26 and H-26'), 1.00 (6H, s, H-27 and H-27'), 0.81 (6H, s, H-29 and H-29'), 0.96 (6H, s, H-30 and H-30'); ^13^C- NMR (CDCl_3_, 150 MHz) *δ*_C_ 32.5 (t, C-1 and C-1'), 26.3 (t, C-2 and C-2'), 77.0 (d, C-3 and C-3'), 40.4 (s, C-4 and C-4'), 42.6 (d, C-5 and C-5'), 18.0 (t, C-6 and C-6'), 31.9 (t, C-7 and C-7'), 40.8 (s, C-8 and C-8'), 54.2 (d, C-9 and C-9'), 36.7 (s, C-10 and C-10'), 127.4 (d, C-11 and C-11'), 125.1 (d, C-12 and C-12'), 137.6 (s, C-13 and C-13'), 42.2 (s, C-14 and C-14'), 24.9 (t, C-15 and C-15'), 31.7 (t, C-16 and C-16'), 49.8 (s, C-17 and C-17'), 130.2 (s, C-18 and C-18'), 40.0 (t, C-19 and C-19'), 32.4 (s, C-20 and C-20'), 36.8 (t, C-21 and C-21'), 35.4 (t, C-22 and C-22'), 71.3 (t, C-23 and C-23'), 17.4 (q, C-24 and C-24'), 18.0 (q, C-25 and C-25'), 16.6 (q, C-26 and C-26'), 19.8 (q, C-27 and C-27'), 172.7 (s, C-28 and C-28'), 24.2 (q, C-29 and C-29'), 32.1 (q, C-30 and C-30'); ESIMS *m/z* 945 [M+Na]^+^; HRESIMS *m/z* 945.6595 [M + Na]^+^ (calcd for C_60_H_90_O_7_Na, 945.6579).

*3α-Hydroxyolean-11,13(18)-dien-28-oic anhydride* (**8**). Yield 45%. white amorphous powder; [*α*]^25^_D_ –84 (*c* 0.2, CHCl_3_); UV (MeOH) *λ*_max_ (log *ε*) 236 (4.08), 248 (3.94), 264 (3.70) nm; IR (KBr) *v*_max_ 3,437, 2,937, 1,800, 1,770, 1,736, 1,457, 1,376, 1,243, 997 cm^−1^; ^1^H-NMR (CDCl_3_, 600 MHz) *δ*_H_ 1.38 (2H, m, H-1*α* and H-1'*α*), 1.63 (2H, m, H-1*β* and H-1'*β*), 1.60 (2H, m, H-2*α* and H-2'*α*), 2.02 (2H, m, H-2*β* and H-2'*β*), 3.44 (2H, br s, H-3 and H-3'), 1.35 (2H, m, H-5 and H-5'), 1.48 (4H, m, H-6 and H-6'), 1.33 (4H, m, H-7 and H-7'), 2.07 (2H, br s, H-9 and H-9'), 5.68 (2H, d, *J* = 10.8 Hz, H-11 and H-11'), 6.42 (2H, dd, *J* = 10.8, 3.0 Hz, H-12 and H-12'), 1.09 (2H, m, H-15*α* and H-15'*α*), 1.61 (2H, m, H-15*β* and H-15'*β*), 2.05 (2H, m, H-16*α* and H-16'*α*), 1.70 (2H, m, H-16*β* and H-16'*β*), 2.53 (2H, d, *J* = 14.4 Hz, H-19*α* and H-19'*α*), 1.78 (2H, d, *J* = 14.4 Hz, H-19*β* and H-19'*β*), 1.31 (2H, m, H-21*α* and H-21'*α*), 1.39 (2H, m, H-21*β* and H-21'*β*), 2.22 (2H, m, H-22*α* and H-22'*α*), 1.45 (2H, m, H-22*β* and H-22'*β*), 0.95 (6H, s, H-23 and H-23'), 0.84 (6H, s, H-24 and H-24'), 0.91 (6H, s, H-25 and H-25'), 0.77 (6H, s, H-26 and H-26'), 0.97 (6H, s, H-27 and H-27'), 0.80 (6H, s, H-29 and H-29'), 0.95 (6H, s, H-30 and H-30'); ^13^C-NMR (CDCl_3_, 150 MHz) *δ*_C_ 32.7 (t, C-1 and C-1'), 25.2 (t, C-2 and C-2'), 76.2 (d, C-3 and C-3'), 37.5 (s, C-4 and C-4'), 48.5 (d, C-5 and C-5'), 18.2 (t, C-6 and C-6'), 32.2 (t, C-7 and C-7'), 40.9 (s, C-8 and C-8'), 54.1 (d, C-9 and C-9'), 36.8 (s, C-10 and C-10'), 127.7 (d, C-11 and C-11'), 125.0 (d, C-12 and C-12'), 137.6 (s, C-13 and C-13'), 42.2 (s, C-14 and C-14'), 24.9 (t, C-15 and C-15'), 31.7 (t, C-16 and C-16'), 49.8 (s, C-17 and C-17'), 130.1 (s, C-18 and C-18'), 40.0 (t, C-19 and C-19'), 32.5 (s, C-20 and C-20'), 36.8 (t, C-21 and C-21'), 35.4 (t, C-22 and C-22'), 28.1 (q, C-23 and C-23'), 21.7 (q, C-24 and C-24'), 17.8 (q, C-25 and C-25'), 16.6 (q, C-26 and C-26'), 19.9 (q, C-27 and C-27'), 172.8 (s, C-28 and C-28'), 24.2 (q, C-29 and C-29'), 32.1 (q, C-30 and C-30'); ESIMS *m/z* 913 [M+Na]^+^; HRESIMS *m/z* 913.6683 [M + Na]^+^ (calcd for C_60_H_90_O_5_Na, 913.6680).

*3α-Acetoxyolean-11,13(18)-dien-28-oic anhydride* (**9**). Yield 47%. colorless needles; [*α*]^25^_D_ –67 (*c* 0.2, CHCl_3_); UV (MeOH) *λ*_max_ (log *ε*) 236 (4.18), 248 (3.98), 265 (3.75) nm; IR (KBr) *v*_max_ 2,935, 1,797, 1,771, 1,733, 1,456, 1,373, 1,241, 998 cm^−1^; ^1^H-NMR (CDCl_3_, 600 MHz) *δ*_H_ 1.26 (2H, m, H-1*α* and H-1'*α*), 1.65 (2H, m, H-1*β* and H-1'*β*), 1.66 (2H, m, H-2*α* and H-2'*α*), 1.96 (2H, m, H-2*β* and H-2'*β*), 4.66 (2H, br s, H-3 and H-3'), 1.29 (2H, m, H-5 and H-5'), 1.49 (4H, m, H-6 and H-6'), 1.35 (4H, m, H-7 and H-7'), 2.05 (2H, br s, H-9 and H-9'), 5.67 (2H, d, *J* = 10.2 Hz, H-11 and H-11'), 6.43 (2H, dd, *J* = 10.2, 2.4 Hz, H-12 and H-12'), 1.11 (2H, m, H-15*α* and H-15'*α*), 1.64 (2H, m, H-15*β* and H-15'*β*), 2.07 (2H, m, H-16*α* and H-16'*α*), 1.72 (2H, m, H-16*β* and H-16'*β*), 2.55 (2H, d, *J* = 15.6 Hz, H-19*α* and H-19'*α*), 1.79 (2H, d, *J* = 15.6 Hz, H-19*β* and H-19'*β*), 1.34 (2H, m, H-21*α* and H-21'*α*), 1.39 (2H, m, H-21*β* and H-21'*β*), 2.22 (2H, m, H-22*α* and H-22'*α*), 1.47 (2H, br d, *J* = 13.8 Hz, H-22*β* and H-22'*β*), 0.85 (6H, s, H-23 and H-23'), 0.89 (6H, s, H-24 and H-24'), 0.93 (6H, s, H-25 and H-25'), 0.78 (6H, s, H-26 and H-26'), 1.01 (6H, s, H-27 and H-27'), 0.81 (6H, s, H-29 and H-29'), 0.96 (6H, s, H-30 and H-30'), 2.09 (3H, s, 3-OAc); ^13^C NMR (CDCl_3_, 150 MHz) *δ*_C_ 33.3 (t, C-1 and C-1'), 22.7 (t, C-2 and C-2'), 78.3 (d, C-3 and C-3'), 36.7 (s, C-4 and C-4'), 49.8 (d, C-5 and C-5'), 18.0 (t, C-6 and C-6'), 32.1 (t, C-7 and C-7'), 40.9 (s, C-8 and C-8'), 54.1 (d, C-9 and C-9'), 36.7 (s, C-10 and C-10'), 127.4 (d, C-11 and C-11'), 125.1 (d, C-12 and C-12'), 137.6 (s, C-13 and C-13'), 42.2 (s, C-14 and C-14'), 24.9 (t, C-15 and C-15'), 31.7 (t, C-16 and C-16'), 49.8 (s, C-17 and C-17'), 130.2 (s, C-18 and C-18'), 40.0 (t, C-19 and C-19'), 32.5 (s, C-20 and C-20'), 36.8 (t, C-21 and C-21'), 35.4 (t, C-22 and C-22'), 27.7 (q, C-23 and C-23'), 21.3 (q, C-24 and C-24'), 17.8 (q, C-25 and C-25'), 16.6 (q, C-26 and C-26'), 19.8 (q, C-27 and C-27'), 172.7 (s, C-28 and C-28'), 24.2 (q, C-29 and C-29'), 32.1 (q, C-30 and C-30'), 170.8 (s, 3-OAc), 21.4 (q, 3-OAc); ESIMS *m/z* 997 [M + Na]^+^; HRESIMS *m/z* 997.6893 [M + Na]^+^ (calcd for C_64_H_94_O_7_Na, 997.6892). Crystal data: C_64_H_94_O_7_·H_2_O (formula weight 993.41), approximate crystal size, 0.64 × 0.56 × 0.30 mm, monoclinic, space group, *P2_1_*, *T* = 110(2) K, *a* = 14.1986(4) Å, *b* = 13.3529(4) Å, *c* = 15.5987(5) Å, *α* = 90°, *β* = 104.518(3)°, *γ* = 90°, *V* = 2862.96(15) Å^3^, *D_c_* = 1.152 Mg/m^3^, *Z* = 2, *F*(000) = 1088, *μ*_(MoK__α)_ = 0.074 mm^−1^. A total of 14049 reflections were collected in the range 2.93° < *θ* < 29.39°, with 9750 independent reflections [*R*_(int)_ = 0.0216], completeness to *θ*_max_ was 99.8%; psi-scan absorption correction applied; full-matrix least-squares refinement on *F*^2^, the number of data/restraints/parameters were 9750/1/681; goodness-of-fit on *F*^2^ = 1.046; final *R* indices [*I* > 2*σ*(*I*)], *R*_1_ = 0.0419, *wR*_2_ = 0.0808; *R* indices (all data), *R*_1_ = 0.0611, *wR*_2_ = 0.0838, absolute structure parameter −0.7(8), largest difference peak and hole, 0.423 and −0.206 e/Å^3^.

### 3.6. Cytotoxicity, Anti-hepatitis B Virus (HBV) Assay and Antibacterial Activity

The experimental details of these assays were carried out according to a previously described procedure [2].

### 3.7. Glucose Uptake Assay

FL83B cells were purchased from the American Type Culture Collection (Rockville, MD, USA) and cultured in F12K medium as described previously [22]. Cells were seeded in 12-well plates (4 × 10^5^ cells/well), cultured overnight, washed with phosphate-buffered saline (PBS, pH 7.4), and subjected to glucose uptake assays in triplicate as described previously with modifications [22,23]. Briefly, each compound was dissolved in DMSO (dimethyl sulfoxide). Cells were incubated in 450 *μ*L of Eagle’s minimum essential medium (MEM) containing 100 nM of insulin, or 20 *μ*M of the indicated compound. In the control, cells were incubated in MEM containing DMSO in a concentration equivalent to that contained in the other groups. At 0, 1, 2, 3, 4, and 5 h, 30 *μ*L of the culture medium was withdrawn and centrifuged at 500 *g* for 5 min. Five microliters of the resulting supernatant was mixed with 250 μL of a glucose assay kit (Glucose GOD FS, DiaSys Diagnostic Systems, Holzheim, Germany) in a 96-well plate and incubated at 37 °C for 10 min. Absorbance at 500 nm was then determined using a microplate reader (Molecular Devices, Sunnyvale, CA, USA). A standard curve was established simultaneously using solutions of glucose in concentrations between 1–10 mM. The data of glucose uptake assays were analysed against the control by two-way analysis of variance (ANOVA), with cell treatment and time (the time points at which medium glucose concentration were measured) as the two parameters. Significance was considered when the p value between groups of cells and the p value of interaction between the two parameters were both < 0.05.

### 3.8. Dual Luciferase Activity Assay

P19 cells (1 × 10^5^ cells/well) were seeded into 24-well dish for overnight. On the next day, cells were transfected with the Wnt reporter construct pGL3-OT (Dr. Bert Vogelstein, the Johns Hopkins University; Addgene No. 16558) and the normalization construct pTK-Renilla, treated with control conditioned medium, Wnt-3a conditioned medium or 1 μM of the compounds (**1**–**5** and **7**–**9**) in Wnt-3a conditioned medium for 16 hours, then the cell lysate was collected and assayed for dual luciferase activities by using Dual Luciferase Reporter Asssay system (Promega). Each assay was done in triplicate. The data of firefly luciferase activity was normalized by that of Renilla luciferase activity. Control conditioned medium and Wnt-3a conditioned medium were prepared as described previously [16].

## 4. Conclusions

After varying their structures at the C-3 and C-28 positions, compounds **1**–**6** revealed greater cytotoxic and antibacterial potential than their parent compounds. Compounds **1**–**5** obviously enhanced glucose uptake of treated cells as compared with untreated cells, and their effects were similar to that of insulin, indicating that compounds **1**–**5** possessed insulin-like hypoglycaemic activities. Compounds **1**, **3**, **5** and **8** at 1 μM of concentration specifically inhibited the Wnt signaling by 20%, 40%, 38% and 32%, respectively. According to our studies, the oleanane-type derivatives endowed with various biological activities and *F*. *polycarpa* identified as a potential plant source for the discovery of promising new drugs. Advanced bioactivity assays and chemical modifications for these compounds will be carried out if sufficient material can be recollected from the plant.
